# Supplementary material for: Asynchronous magnetic resonance elastography: Shear wave speed reconstruction using noise correlation of incoherent waves
Source: Magn Reson Med. 2022 Oct 27;89(3):990–1001. doi: 10.1002/mrm.29502 (PMC9792433; doi:10.1002/mrm.29502)
Supplement: Supplementary file 1 — DATA S1 MATLAB code used to generate the numerical simulations of Figure 1 [file MRM-89-990-s002.zip › k-Wave/helpfiles/stackedPlot.html]

stackedPlot :: Functions (k-Wave)


# stackedPlot

Stacked linear plot.

## Syntax

```
stackedPlot(data)
stackedPlot(x, data)
stackedPlot(x, y, data)
```

## Description

`stackedPlot` produces a series of stacked linear plots of the rows in `data` (a 2D matrix) against the vector `x`. The vector `y` defines the y-axis label for each linear plot. The plot scaling is defined using the global maximum and minimum of the data. The plot can be annotated in the normal fashion after display. For example, each of the following

```
stackedPlot(rand(5, 100));
stackedPlot(0.1:0.1:10, rand(5, 100));
stackedPlot(0.1:0.1:10, {'a', 'b', 'c', 'd', 'e'}, rand(5, 100));
```

will produce stacked plots of random data, but with different axis labels.

## Inputs

|  |  |
| --- | --- |
| `x` | vector defining the x-axis values |
| `y` | vector defining the y-axis labels used for each plot |
| `data` | 2D matrix to plot |

## Optional Inputs

Optional 'string', value pairs that may be used to modify the default computational settings.

| Input | Valid Settings | Default | Description |
| --- | --- | --- | --- |
| `'Spacing'` | *(numeric scalar)* | `0.1` | Spacing between the individual plots as a fraction of the maximum plot range. |
| `'Symmetric'` | *(Boolean scalar)* | `false` | Boolean controlling whether the max and minimum of the individual plots are forced to be symmetric. This forces the y-ticks to be at zero. |

## See Also

`plot`
